# Effects of neuromuscular electrical stimulation training on muscle size in collegiate track and field athletes

**DOI:** 10.1371/journal.pone.0224881

**Published:** 2019-11-13

**Authors:** Taku Wakahara, Ayumu Shiraogawa

**Affiliations:** 1 Faculty of Health and Sports Science, Doshisha University, Kyotanabe, Kyoto, Japan; 2 Human Performance Laboratory, Waseda University, Tokorozawa, Saitama, Japan; Victoria University, AUSTRALIA

## Abstract

The purpose of this study was to examine the effects of neuromuscular electrical stimulation training for 12 weeks on the abdominal muscle size in trained athletes. Male collegiate track and field athletes participated in the present study and were randomly allocated to either training or control groups. Eleven participants of the training group completed a 60-session training program over a 12-week period (23 min/session, 5 days/week) involving neuromuscular electrical stimulation (mostly 20 Hz) for the abdominal muscles in addition to their usual training for the own events. The participants of the control group (n = 13) continued their usual training. Before and after the intervention period, cross-sectional areas of the rectus abdominis and abdominal oblique muscles (the internal and external obliques and transversus abdominis) and subcutaneous fat thickness were measured with magnetic resonance and ultrasound imaging. There were no significant changes in cross-sectional area of the rectus abdominis or abdominal oblique muscles or in subcutaneous fat thickness in the training or control groups after the intervention period. The change in cross-sectional area of the rectus abdominis in each participant was not significantly correlated with pre-training cross-sectional area and neither was the mean value of fat thickness at pre- and post-training. These results suggest that low-frequency (20 Hz) neuromuscular electrical stimulation training for 12 weeks is ineffective in inducing hypertrophy of the abdominal muscles in trained athletes, even when they have a thin layer of subcutaneous fat.

## Introduction

Neuromuscular electrical stimulation (NMES) evokes local muscular contractions and can be applied as a stimulus for training. Previous studies have demonstrated that NMES training induces an increase in muscular strength during voluntary contraction even after relatively short training periods (3–6 weeks) [1]. In addition, NMES training increased voluntary muscle activation as evaluated by twitch interpolation [2] and surface electromyography [3]. Therefore, NMES training has been considered to increase voluntary muscular strength in a short period of time through neural adaptations [4].

The effect of NMES training on muscle hypertrophy is equivocal in the literature. Some studies found a significant increase in size at the whole muscle [5–8] and fiber levels [8–11] after NMES training, while others did not observe a hypertrophic change at the whole muscle [12,13] or fiber level [14–16]. Among the studies, 8 to 9 weeks of NMES training induced significant hypertrophy [5–8,11], whereas less than 6 weeks of training resulted in insignificant changes in muscle size [12–15]. Thus, the inconsistent results regarding hypertrophic adaptation after NMES training may be due to the length of the training period and, therefore, additional studies with a sufficient length of the training period are required to clarify the effect of NMES training on muscular hypertrophy.

The NMES training could bring a benefit for competitive athletes [17]. Previous studies reported significant gains in muscular strength in athletes after NMES training [1,18]. However, the effects of NMES training on the muscle size of trained athletes are poorly understood. St. Pierre et al. [19] investigated the effect of 8 days of NMES training on muscle fiber size of the vastus lateralis in college athletes (seven male football players and three female volleyball players). They reported a significant decrease in the cross-sectional area (CSA) of type II fibers in the male athletes after NMES training, whereas no significant change was observed in the CSA of type I fibers in the male athletes or the CSAs of either type I or type II fibers in the female athletes. Delitto et al. [20] examined the effect of NMES training for the quadriceps femoris of an elite male weightlifter while he continued his regular training. They found a significant increase in the CSA of type I fibers of the vastus lateralis and a significant decrease in the CSA of type IIa and IIb fibers after an initial 4 weeks of NMES training. Unfortunately, the length of the training periods was relatively short and the number of participants was small in these studies. Thus, the effects of NMES training on muscle size in trained athletes have yet to be investigated.

Generally, athletes have less body fat and larger muscles compared to untrained individuals. Because fat has high electrical resistance, its thickness is negatively correlated with current delivered into muscle for a given electrical stimulus applied to the skin [21]. Hence, athletes who have a thin fat layer could be expected to be highly responsive to NMES. Meanwhile, it may be difficult to elicit a hypertrophic response in trained athletes who already have large muscles, because of a “ceiling effect” [22]. The purpose of the present study was to examine whether NMES training induces muscle hypertrophy in trained athletes. To this end, we investigated the effect of NMES training over a 12-week period on the abdominal muscle size in collegiate track and field athletes. The abdominal muscles were chosen because these muscles were reported to be greater in track and field athletes than untrained individuals [23,24].

## Materials and methods

### Participants

The required sample size for the present study was calculated with G*Power software. As mentioned above, little information is available regarding the effects of NMES training on the muscles size in athletes. Thus, we used data from a previous study that reported hypertrophy of the rectus abdominis (RA) in male collegiate athletes [25]. As a result, at least 11 participants were required to obtain a 0.97 effect size [25], with an α level of 0.05, and a power (1 − β) of 0.80. According to the calculation, twenty-six male collegiate athletes were recruited from a university track and field club in April to June 2017, and randomly assigned to either training (n = 13) or control (n = 13) groups. They were regional to intercollegiate athletes and competed in the following disciplines: sprint running (100, 200, and 400 m, n = 8); hurdling (110 and 400 m, n = 6); middle-distance running (800 m, n = 2); jumping (long jump, triple jump, and pole vault, n = 6); javelin throwing (n = 3); and decathlon (n = 1). Endurance runners who specialize in races over 1500 m, race walkers or female athletes were not recruited in the present study. One participant of the training group (a sprint runner) did not take part in the measurement process after the training period due to a scheduling conflict. Another participant of the training group (a middle-distance runner) was unable to hold his breath during magnetic resonance (MR) image recording (see below). Thus, we analyzed the data of 11 participants of the training group (age: 19.3 ± 0.8 years, height: 175.0 ± 4.5 cm, body mass: 67.6 ± 4.5 kg, mean ± standard deviation [*SD*]) and 13 participants of the control group (19.7 ± 0.8 years, 174.8 ± 5.7 cm, 67.7 ± 7.1 kg). They ranged in age from 18 to 20 years, and had, on average, 7.8 ± 2.7 years of experience in track and field. There were no significant differences between the groups in their baseline physical characteristics (age, height, or body mass). All the participants were fully informed of the purpose and potential risks involved in this study, and they gave their written informed consent. The present study was approved by the Doshisha University Research Ethics Review Committee regarding Human Subject Research (number: 16072) and was registered in the University Hospital Medical Information Network Clinical Trial Registry (UMIN-CTR, UMIN000026026).

### NMES training

Participants of the training group trained their abdominal muscles with a portable battery-powered NMES device (Sixpad Abs Fit, MTG, Japan). The device was attached over their RA with six self-adhesive electrodes (64 mm × 37 mm) and was fastened by an elastic band. In the training sessions, biphasic square wave pulses (100 μs) were delivered at 2–20 Hz (mostly 20 Hz) for 23 min in a preprogrammed manner. The participants selected the maximum stimulation intensity (from level 1 to 15. The level 15 corresponds to 3.8 mArms) that they could tolerate in each training session. The training program consisted of 60 NMES sessions over a 12-week period with a frequency of 5 days per week. The participants trained at times and places of their own choosing, but were instructed not to rest on two consecutive days. They were required to maintain a training log that included the day, time, and intensity of NMES. Both groups were instructed to continue their usual training for the own events and their normal daily habits except for the addition of NMES training in the training group. The present study was conducted during a competitive season of track and field (from June to September).

### MR imaging

**Fig 1:**
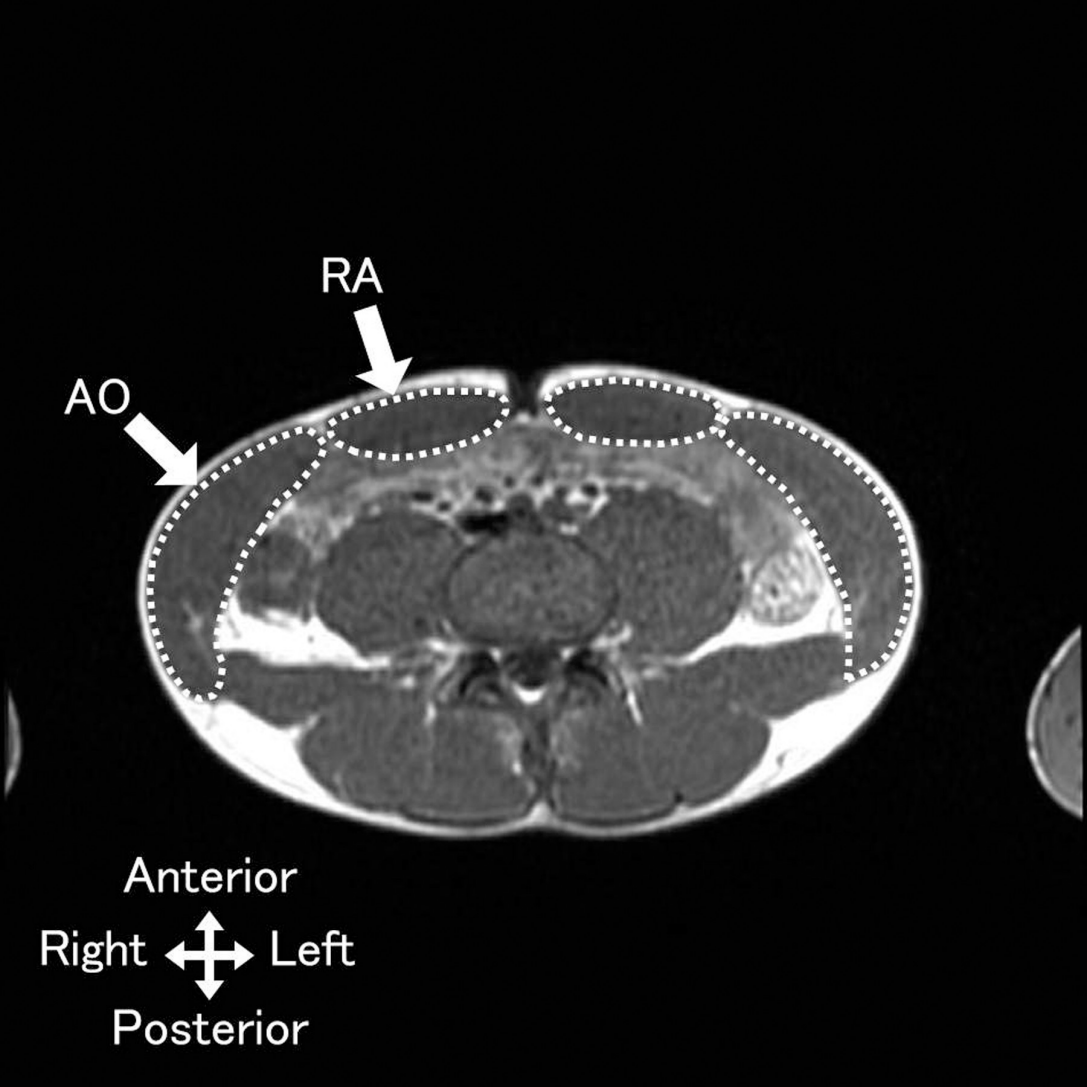
A typical magnetic resonance image of the trunk. The white broken lines are the borders of muscles. RA, rectus abdominis; AO, abdominal oblique muscles (internal and external obliques and transversus abdominis).

A series of transverse MR images of the lower trunk were recorded before and after the intervention period with a 1.5-T MR scanner (Echelon Vega, Hitachi Medical Corporation, Japan) using a 16-channel body array coil (Fig 1, echo time: 8.8 ms; repetition time: 500 ms; matrix: 256 × 192; field of view: 360 mm; slice thickness: 6 mm; gap: 4 mm). The participants lay supine in the magnet bore with their arms and legs extended. During the scan, they were required to hold their breath for approximately 20 s to reduce motion artifacts caused by their respiration. Scanning was performed twice to cover the superior and inferior regions to the umbilicus. Scanned images were reconstructed to a matrix size of 512 × 512 and transferred to a computer. In the images, CSAs of RA and the abdominal oblique muscles (internal and external obliques and transversus abdominis [AO]) were measured for each side (right and left) using dedicated software (Image J, National Institute of Health, USA). The RA of each side consists of several bellies, which are divided by tendinous intersections. The superior–inferior position of the tendinous intersections was different among participants and between sides. Thus, RA CSA was initially measured for several slices around the superior border of the pelvis. Thereafter, the peak CSA value nearest to the border was selected at each side. The AO CSA was measured at the superior border of the pelvis [26]. The tracing was performed twice for each slice by a person who was blinded to the group allocation. The mean of the two traced values was calculated, and the overall mean of the left and right sides of each muscle was used for subsequent analyses. The mean coefficient of variation (CV) for the two CSA measurements was 0.8 ± 0.5% and 0.4 ± 0.3% for RA and AO, respectively. Intraclass correlation coefficient (ICC {1, 2}) of the measurements was 0.999 for both RA and AO.

### Ultrasound imaging

**Fig 2:**
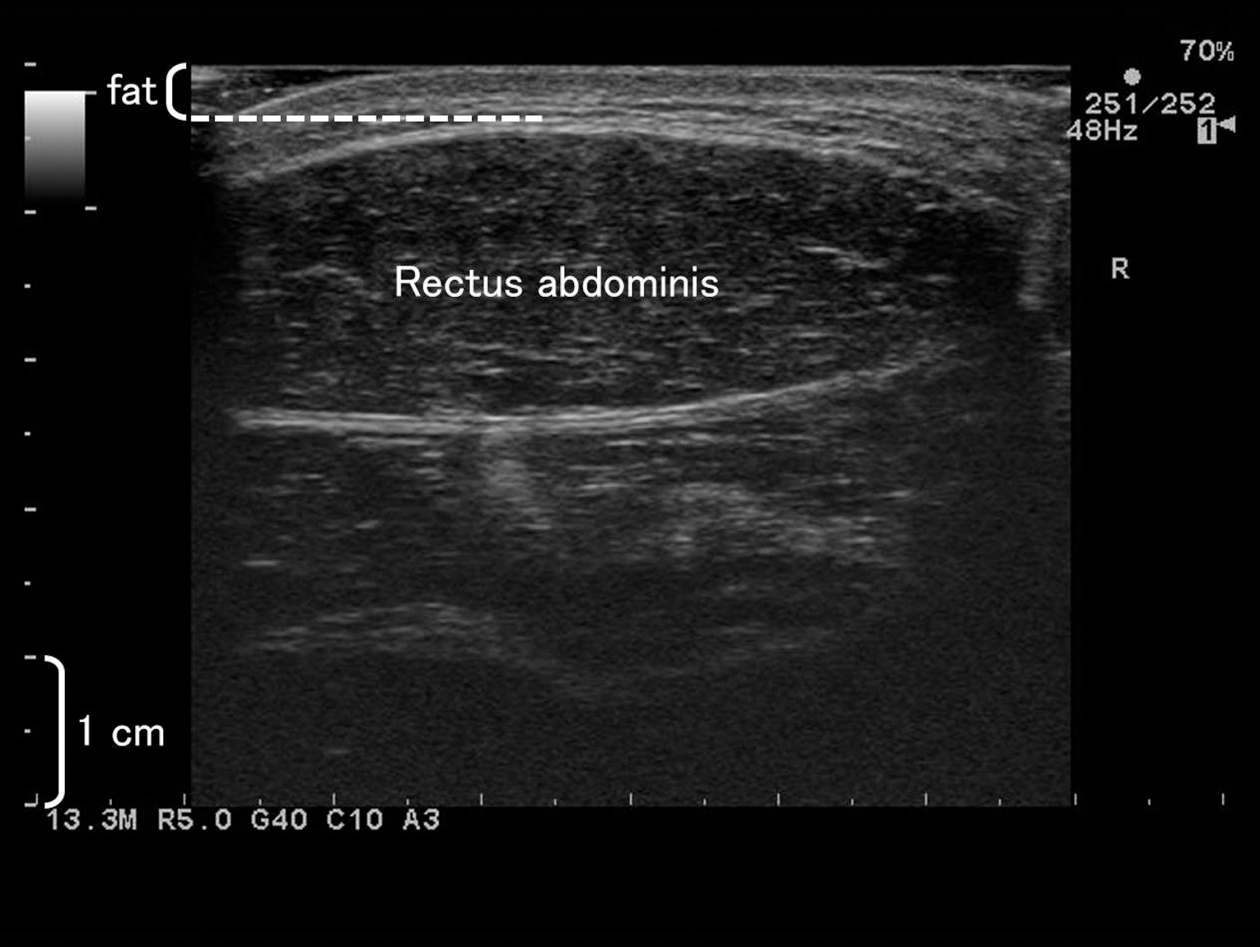
A typical ultrasound image of the abdomen. The white broken line shows the border between subcutaneous fat and the rectus abdominis.

An ultrasonic apparatus (ProSound α7, Hitachi Aloka Medical, Japan) with a 6-cm linear array probe (UST-5712, Hitachi Aloka Medical, Japan) was used to record cross-sectional images of both sides of the abdomen (Fig 2). The probe with water-soluble transmission gel was attached to the skin at the belly of RA nearest to the umbilicus while the participants lay supine on a bed. Ultrasound scanning was repeated three times for each side. In the scanned images, the thickness of the subcutaneous fat was measured as a distance from the skin to the border between subcutaneous fat and muscle [27] using Image J software (National Institute of Health, USA). The overall mean of the subcutaneous fat thickness (three values for each side, right and left) was calculated and used for further analyses. In addition, the mean of the fat thickness at pre- and post-training was calculated as a representative value of fat thickness over the training period. The mean CV and ICC {1, 3} of the measurements was 3.8 ± 2.2% and 0.997, respectively.

### Statistics

A two-way analysis of variance (ANOVA) was used to analyze the effects of time (before and after the intervention period) and group (training and control groups) on muscle CSAs, subcutaneous fat, and body mass. The partial η^2^ was calculated as an index of effect size of ANOVA. The relationship between two variables was tested using Pearson’s product moment correlation coefficient. These analyses were performed using a statistical software package (IBM SPSS Statistics, version 25, USA). Statistical significance for the tests was set at *P* < 0.05.

## Results

**Fig 3:**
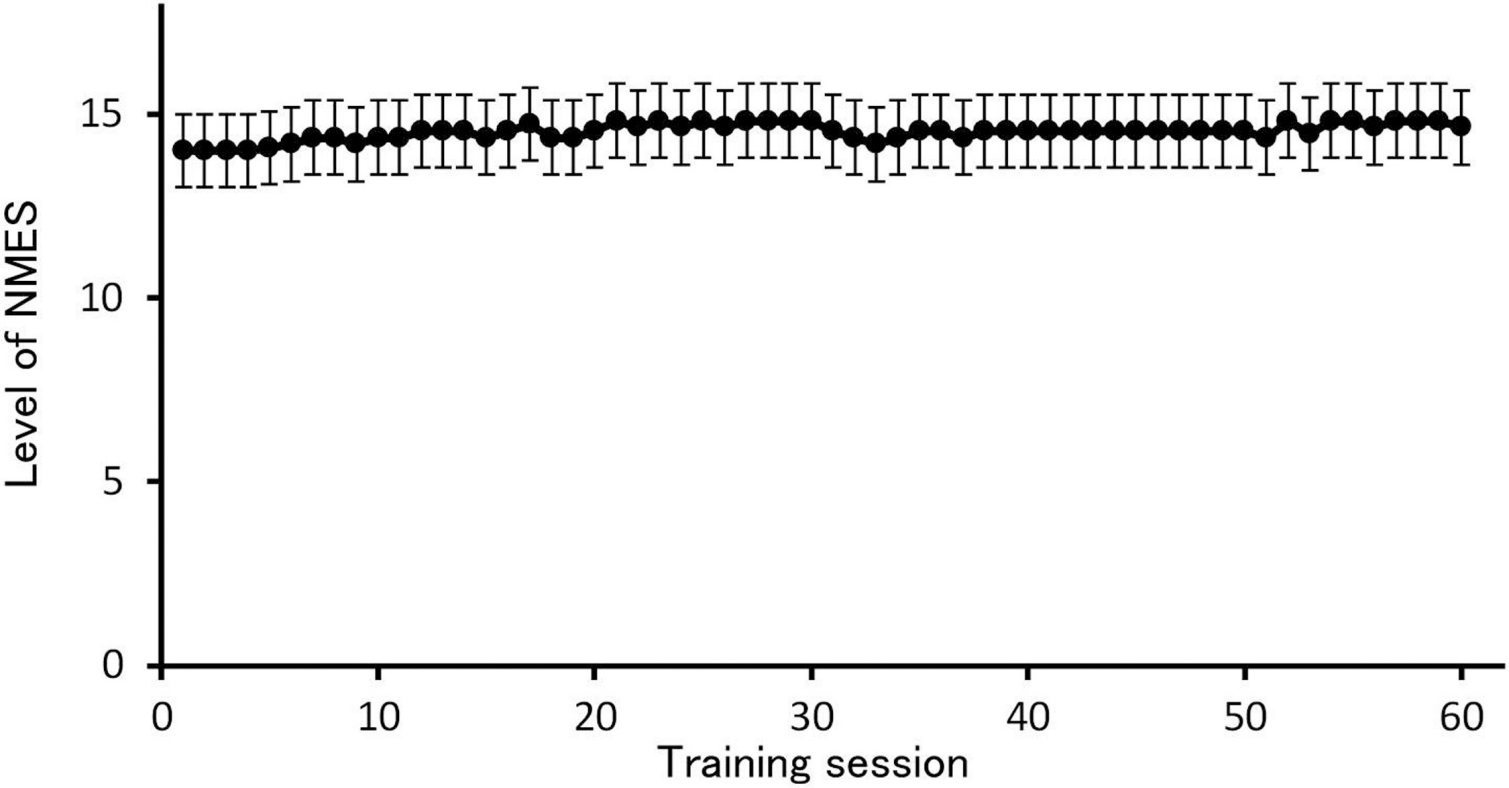
The level of neuromuscular electrical stimulation in each training session. The participants selected the maximum stimulation intensity that they could tolerate for each session. Intensity levels ranged from 1 to 15. NMES, neuromuscular electrical stimulation.

According to the training log, all the participants of the training group (n = 11) completed 60 NMES training sessions. However, two of the participants took two consecutive rest days during the training period. Almost all participants were able to use the maximum limit of the device (level 15) within the first week of the training intervention (Fig 3). The mean NMES level for each participant over the training period was 12.0–15.0. No participant reported serious discomfort using NMES.

Table 1 shows the mean and *SD* of RA and AO CSAs and fat thickness. Two-way ANOVA showed no significant main effects for time (*P* = 0.917, partial η^2^ = 0.001) or group (*P* = 0.983, partial η^2^ < 0.001) on RA CSA with no significant interaction of the two factors (*P* = 0.060, partial η^2^ = 0.151). Similarly, no significant main effects for time (*P* = 0.308, partial η^2^ = 0.047) or group (*P* = 0.303, partial η^2^ = 0.048) were found on AO CSA, also with no significant interaction of the two factors (*P* = 0.641, partial η^2^ = 0.010). There were no significant main effects for time (*P* = 0.762, partial η^2^ = 0.004) or group (*P* = 0.448, partial η^2^ = 0.026) on fat thickness with no significant interaction of the two factors (*P* = 0.400, partial η^2^ = 0.032). No significant main effects for time (*P* = 0.554, partial η^2^ = 0.016) or group (*P* = 0.989, partial η^2^ < 0.001) were found on body mass with no significant interaction of the two factors (*P* = 0.878, partial η^2^ = 0.001).

**Table 1:** Abdominal muscle cross-sectional area, fat thickness, and body mass before and after the intervention period.

	Training group						Control group					
	Before			After			Before			After		
RA CSA (cm^2^)	8.4	±	0.8	8.6	±	1.1	8.6	±	2.3	8.4	±	2.1
AO CSA (cm^2^)	26.8	±	4.3	26.7	±	3.3	25.3	±	3.4	24.9	±	3.9
Fat thickness (cm)	0.44	±	0.12	0.42	±	0.10	0.50	±	0.26	0.51	±	0.32
Body mass (kg)	67.6	±	4.5	67.8	±	4.0	67.7	±	7.1	67.8	±	7.6

Data are presented as mean ± standard deviation. RA, rectus abdominis; CSA, cross-sectional area; AO, abdominal oblique muscles.

**Fig 4:**
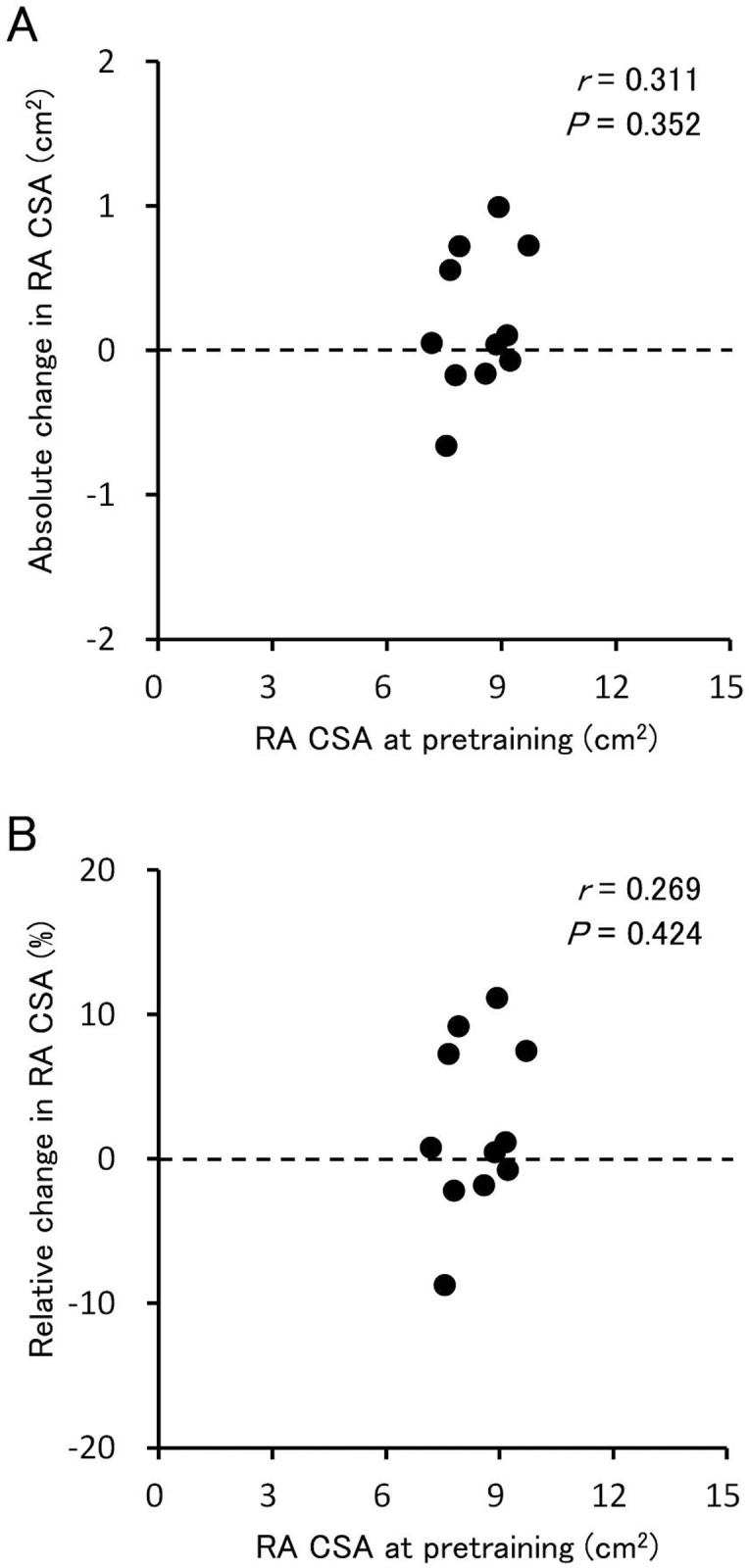
The relationship between cross-sectional area of the rectus abdominis at pretraining and its changes after training. RA, rectus abdominis; CSA, cross-sectional area. (A) The relationship between RA CSA at pretraining and the absolute change in the CSA. (B) The relationship between RA CSA at pretraining and the relative change in the CSA.

**Fig 5:**
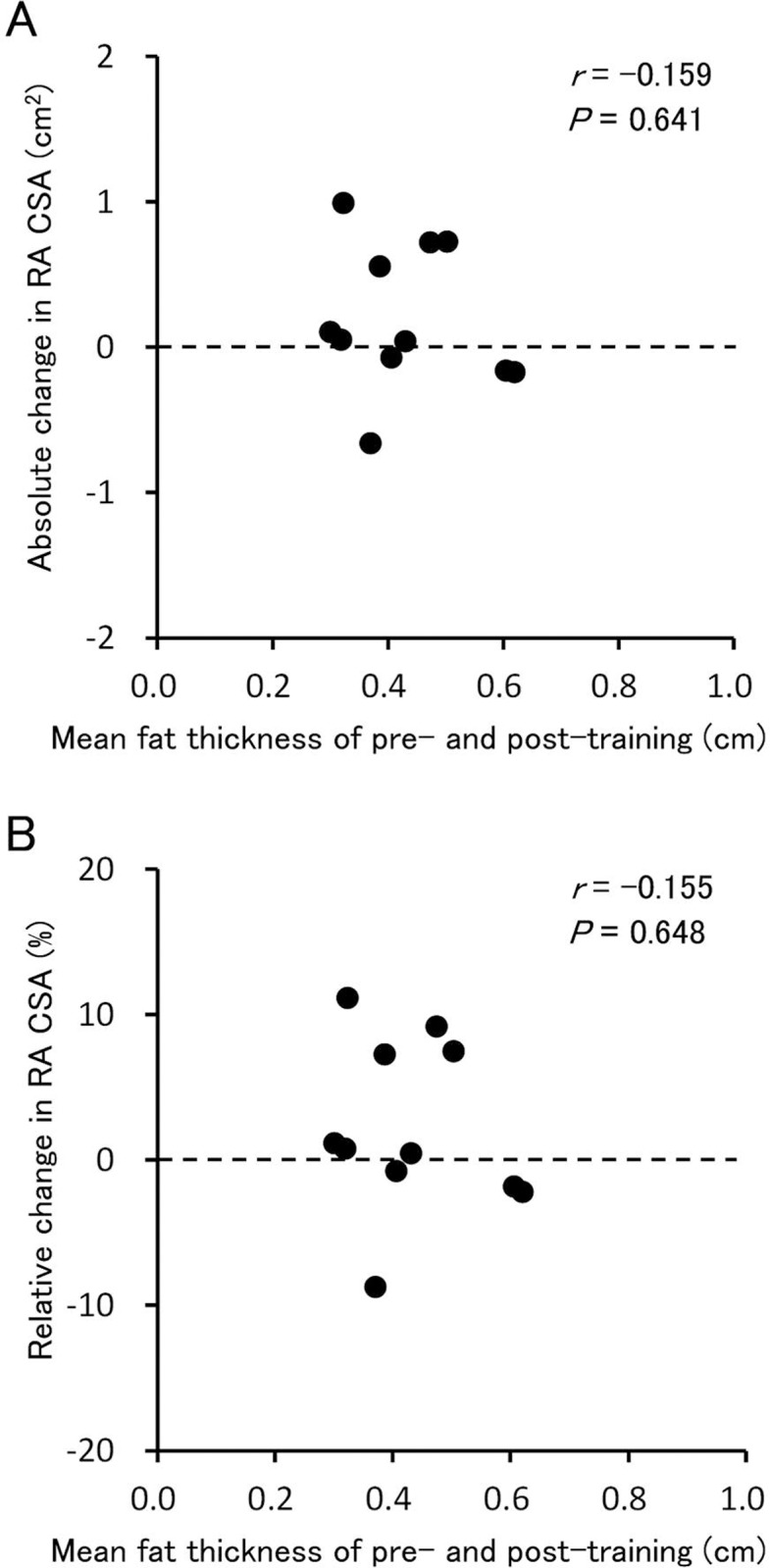
The relationship between mean fat thickness of pre- and post-training and changes in cross-sectional area of the rectus abdominis after training. RA, rectus abdominis; CSA, cross-sectional area. (A) The relationship between mean fat thickness and the absolute change in RA CSA. (B) The relationship between mean fat thickness and the relative change in the CSA.

The RA CSA at pretraining was not significantly correlated with its absolute (*r* = 0.311, *P* = 0.352) or relative (*r* = 0.269, *P* = 0.424) changes during the training period (Fig 4). In addition, the mean subcutaneous fat thickness of pre- and post-training values was not significantly correlated with the absolute (*r* = −0.159, *P* = 0.641) or relative (*r* = −0.155, *P* = 0.648) changes in the RA CSA (Fig 5).

## Discussion

In the present study, CSAs of RA and AO did not significantly change after 12 weeks of NMES training in male collegiate track and field athletes. Although some previous studies [19,20] have investigated the effect of NMES training on muscle size in athletes, the findings were inconclusive. This was partly because the number of training sessions and length of the training periods in these studies (seven sessions in 8 days [19] and 12 sessions in 4 weeks [20]) were not sufficient to induce a detectable change in muscle size. Taking this into account, we designed a NMES training program that consisted of 60 sessions over 12 weeks. Nonetheless, no significant change was observed in CSAs of RA or AO in the trained athletes after the training period. These results suggest that NMES training at low frequency (20 Hz) is ineffective in inducing hypertrophy of the abdominal muscles in trained athletes.

The mean fat thickness over the RA at pre- and post-training was 0.43 ± 0.11 cm in the training group. This value is less than one-third of the thickness (1.5 cm) previously determined in 51 untrained men aged 20–29 years [28] using a method similar to that followed in the present study. Because fat is an electrically high-resistant tissue [21], its thickness has a negative impact on the current delivered into the muscle underlying the fat layer. In fact, there was a strong negative correlation between fat thickness of the thigh and electrical current into the quadriceps femoris measured with needle electrodes for a given stimulus applied to the skin [21]. Therefore, a thin layer of fat would have allowed for an effective NMES delivery into the abdominal muscles of the study participants. However, no significant change was observed in the RA CSA after 12 weeks of NMES training. In addition, there was no significant correlation between mean fat thickness and absolute or relative changes in the RA CSA, although this might be related to the relatively small sample size and a thin fat layer in all the participants. Taken together, the lack of muscle hypertrophy despite the thin layer of fat further suggests an unresponsiveness of athletes’ muscles to NMES training.

A plausible explanation for the lack of hypertrophy is the training status of the participants. The pre-training value of RA CSA in the training group (8.4 ± 0.8 cm^2^) was approximately 25% greater than the previously reported value (6.7 ± 1.9 cm^2^) in 23 young sedentary men [29]. This implies that the RA of the participants in the training group had already hypertrophied to some extent by their competitive experience in track and field, especially in strength-oriented events such as sprint running and jumping. Generally, it is difficult to induce muscle hypertrophy in strength-trained athletes, because of a “ceiling effect” [22]. For example, Häkkinen et al. [30] reported that muscle fiber CSA of the vastus lateralis in elite weightlifters was not significantly increased after a 1-year training period. Thus, the large RA of our participants at pre-training might have limited the hypertrophic adaptation by NMES training. However, RA CSA at pretraining was not correlated with its change by training intervention. Therefore, the lack of hypertrophic response in the RA may not be solely explained by the training status of the participants.

Other factors that may explain the lack of hypertrophy include the NMES parameters, although Maffiuletti [2] pointed out that the effectiveness of a NMES training program does not rely, for the most part, on NMES parameters, but more to the characteristics of the participants. A systematic review of NMES training showed that, in healthy people, high-frequency (> 50 Hz) NMES training resulted in an increase in muscle size, whereas the effect of low-frequency (< 20 Hz) NMES training on muscle size was conflicting [31]. Hence, the low frequency (mostly 20 Hz) of the present NMES device may lead to the insignificant changes in the abdominal muscle size. However, Nishikawa et al. [32] observed a significant increase in thickness of the vastus lateralis of elderly women after 8 weeks of NMES training using a device which stimulate the quadriceps femoris at the same frequency to the present one (manufactured by the same company). Therefore, the lack of hypertrophy cannot be solely explained by the frequency of NMES. Meanwhile, the participants of the training group were requested to select the maximal tolerable level of NMES. The selected levels were near the limit of the device during the first week of the training period. This was inconsistent with earlier reports that showed a gradual increase of the maximal tolerable level of NMES during training periods [8,33]. It is possible that the limit level of the device used in the present study may not have reached the actual maximum tolerable stimulation of the participants. It has been shown that the magnitude of hypertrophy of the quadriceps femoris was significantly greater in high-intensity NMES training (62.5% of maximal voluntary contraction) than in low-intensity ones (32.6% of maximal voluntary contraction) in untrained young men (Natsume et al. 2018). Although Natsume et al. [8] also reported that even low-intensity NMES training over 8 weeks resulted in significant hypertrophy in both whole muscle and fiber levels, the intensity of NMES might be an important factor to elicit muscle hypertrophy especially in trained athletes.

The major limitation of the present study is that we did not control the practice or training other than NMES training. It has been shown that the magnitude of muscle fiber hypertrophy induced by resistance training is interfered by the addition of endurance training [34,35]. In this regard, Kikuchi et al. [36] compared hypertrophy of elbow flexors after 8 weeks of arm curl training alone and arm curls combined with sprint interval training on a cycle ergometer. They reported that the magnitude of muscle hypertrophy was lower in the concurrent resistance and sprint interval training when compared with resistance training alone. This finding suggests that the interference effect may exist even when the intensity of endurance training is high and when the target muscle of the endurance training is not the same as that of the resistance training. Although endurance runners who specialize in races over 1500 m were not recruited in the present study, the practice and training for their own events might have interfered with a possible hypertrophic response of the abdominal muscles by NMES training.

## Conclusions

In summary, the present results demonstrated that 12 weeks of NMES training using a Sixpad Abs Fit device resulted in insignificant changes in the CSA of abdominal muscles in collegiate track and field athletes. The results suggest that low-frequency (20 Hz) NMES training is ineffective in inducing muscle hypertrophy of the abdominal muscles in trained athletes even when they have a thin subcutaneous fat layer.

## Supporting information

S1 Table Abdominal muscle cross-sectional area, fat thickness, and body mass of each participant before and after the intervention period. RA, rectus abdominis; CSA, cross-sectional area; AO, abdominal oblique muscles
(XLSX)

## References

[1] Filipovic A, Kleinöder H, Dörmann U, Mester J. Electromyostimulation—a systematic review of the effects of different electromyostimulation methods on selected strength parameters in trained and elite athletes. J Strength Cond Res. 2012;26: 2600–2614. 10.1519/JSC.0b013e31823f2cd1 22067247

[2] Maffiuletti N, Pensini M, Martin A. Activation of human plantar flexor muscles increases after electromyostimulation training. J Appl Physiol. 2002;92: 1383–1392. 10.1152/japplphysiol.00884.2001 11896001

[3] Gondin J, Duclay J, Martin A. Soleus- and gastrocnemii-evoked V-wave responses increase after neuromuscular electrical stimulation training. J Neurophysiol. 2006;95: 3328–3335. 10.1152/jn.01002.2005 16481458

[4] Hortobágyi T, Maffiuletti NA. Neural adaptations to electrical stimulation strength training. Eur J Appl Physiol. 2011;111: 2439–2449. 10.1007/s00421-011-2012-2 21643920 PMC3175340

[5] Ruther CL, Golden CL, Harris RT, Dudley GA. Hypertrophy, resistance training, and the nature of skeletal muscle activation. J Strength Cond Res. 1995;9: 155–159.

[6] Stevenson SW, Dudley GA. Dietary creatine supplementation and muscular adaptation to resistive overload. Med Sci Sports Exerc. 2001;33: 1304–1310. 10.1097/00005768-200108000-00010 11474331

[7] Gondin J, Guette M, Ballay Y, Martin A. Electromyostimulation training effects on neural drive and muscle architecture. Med Sci Sports Exerc. 2005;37: 1291–1299. 10.1249/01.mss.0000175090.49048.41 16118574

[8] Natsume T, Ozaki H, Kakigi R, Kobayashi H, Naito H. Effects of training intensity in electromyostimulation on human skeletal muscle. Eur J Appl Physiol. 2018;118: 1339–1347. 10.1007/s00421-018-3866-3 29679248

[9] Cabric M, Appell HJ, Resic A. Fine structural changes in electrostimulated human skeletal muscle. Evidence for predominant effects on fast muscle fibres. Eur J Appl Physiol Occup Physiol. 1988;57: 1–5. 10.1007/bf00691229 3342785

[10] Pérez M, Lucia A, Rivero JL, Serrano AL, Calbet JA, Delgado MA, et al Effects of transcutaneous short-term electrical stimulation on M. vastus lateralis characteristics of healthy young men. Pflugers Arch. 2002;443: 866–874. 10.1007/s00424-001-0769-6 11889587

[11] Gondin J, Brocca L, Bellinzona E, D'Antona G, Maffiuletti NA, Miotti D, et al Neuromuscular electrical stimulation training induces atypical adaptations of the human skeletal muscle phenotype: a functional and proteomic analysis. J Appl Physiol. 2011;110: 433–450. 10.1152/japplphysiol.00914.2010 21127206

[12] Singer KP. The influence of unilateral electrical muscle stimulation on motor unit activity patterns in atrophic human quadriceps. Aust J Physiother. 1986;32: 31–37. 10.1016/S0004-9514(14)60641-3 25026319

[13] Martin L, Cometti G, Pousson M, Morlon B. Effect of electrical stimulation training on the contractile characteristics of the triceps surae muscle. Eur J Appl Physiol Occup Physiol. 1993;67: 457–461. 10.1007/bf00376463 8299618

[14] Eriksson E, Häggmark T, Kiessling KH, Karlsson J. Effect of electrical stimulation on human skeletal muscle. Int J Sports Med. 1981;2: 18–22. 10.1055/s-2008-1034578 7333731

[15] Kim CK, Takala TE, Seger J, Karpakka J. Training effects of electrically induced dynamic contractions in human quadriceps muscle. Aviat Space Environ Med. 1995;66: 251–255. 7661836

[16] Thériault R, Boulay MR, Thériault G, Simoneau JA. Electrical stimulation-induced changes in performance and fiber type proportion of human knee extensor muscles. Eur J Appl Physiol Occup Physiol. 1996;74: 311–317. 10.1007/bf02226926 8911822

[17] Maffiuletti NA. Physiological and methodological considerations for the use of neuromuscular electrical stimulation. Eur J Appl Physiol. 2010;110: 223–234. 10.1007/s00421-010-1502-y 20473619

[18] Filipovic A, Kleinöder H, Dörmann U, Mester J. Electromyostimulation—a systematic review of the influence of training regimens and stimulation parameters on effectiveness in electromyostimulation training of selected strength parameters. J Strength Cond Res. 2011;25: 3218–3238. 10.1519/JSC.0b013e318212e3ce 21993042

[19] St Pierre D, Taylor AW, Lavoie M, Sellers W, Kots YM. Effects of 2500 Hz sinusoidal current on fibre area and strength of the quadriceps femoris. J Sports Med Phys Fitness. 1986;26: 60–66. 2940419

[20] Delitto A, Brown M, Strube MJ, Rose SJ, Lehman RC. Electrical stimulation of quadriceps femoris in an elite weight lifter: a single subject experiment. Int J Sports Med. 1989;10: 187–191. 10.1055/s-2007-1024898 2674035

[21] Petrofsky J. The effect of the subcutaneous fat on the transfer of current through skin and into muscle. Med Eng Phys. 2008;30: 1168–1176. 10.1016/j.medengphy.2008.02.009 18400550

[22] Schoenfeld B. Factors in maximal hypertrophic development In: Schoenfeld B. Science and development of muscle hypertrophy. Champaign USA: Human Kinetics; 2016 pp. 105–113.

[23] Abe T, Kumagai K, Brechue WF. Fascicle length of leg muscles is greater in sprinters than distance runners. Med Sci Sports Exerc. 2000;32: 1125–1129. 10.1097/00005768-200006000-00014 10862540

[24] Tanaka NI, Komuro T, Tsunoda N, Aoyama T, Okada M, Kanehisa H. Trunk muscularity in throwers. Int J Sports Med. 2013;34: 56–61. 10.1055/s-0032-1316316 22903318

[25] Takai Y, Nakatani M, Akamine T, Shiokawa K, Komori D, Kanehisa H. Effect of core training on trunk flexor musculature in male soccer players. Sports Med Int Open. 2017;1: E147–E154. 10.1055/s-0043-115377 30539100 PMC6226075

[26] Kubo J, Ohta A, Takahashi H, Kukidome T, Funato K. The development of trunk muscles in male wrestlers assessed by magnetic resonance imaging. J Strength Cond Res. 2007;21: 1251–1254. 10.1519/R-19815.1 18076225

[27] Abe T, Kondo M, Kawakami Y, Fukunaga T. Prediction equations for body composition of Japanese adults by B-mode ultrasound. Am J Hum Biol. 1994;6: 161–170. 10.1002/ajhb.1310060204 28548275

[28] Kanehisa H, Miyatani M, Azuma K, Kuno S, Fukunaga T. Influences of age and sex on abdominal muscle and subcutaneous fat thickness. Eur J Appl Physiol. 2004;91: 534–537. 10.1007/s00421-003-1034-9 14735364

[29] Tanaka NI, Yamada M, Tanaka Y, Fukunaga T, Nishijima T, Kanehisa H. Difference in abdominal muscularity at the umbilicus level between young and middle-aged men. J Physiol Anthropol. 2007;26: 527–532. 18092508 10.2114/jpa2.26.527

[30] Häkkinen K, Komi PV, Alén M, Kauhanen H. EMG, muscle fibre and force production characteristics during a 1 year training period in elite weight-lifters. Eur J Appl Physiol Occup Physiol. 1987;56: 419–427. 10.1007/bf00417769 3622485

[31] Sillen MJ, Franssen FM, Gosker HR, Wouters EF, Spruit MA. Metabolic and structural changes in lower-limb skeletal muscle following neuromuscular electrical stimulation: a systematic review. PLoS One. 2013;8: e69391 10.1371/journal.pone.0069391 24019860 PMC3760845

[32] Nishikawa Y, Watanabe K, Kawade S, Takahashi T, Kimura H, Maruyama H, et al The effect of a portable electrical muscle stimulation device at home on muscle strength and activation patterns in locomotive syndrome patients: A randomized control trial. J Electromyogr Kinesiol. 2019;45: 46–52. 10.1016/j.jelekin.2019.02.007 30802718

[33] Alon G, McCombe SA, Koutsantonis S, Stumphauzer LJ, Burgwin KC, Parent MM, et al Comparison of the effects of electrical stimulation and exercise on abdominal musculature. J Orthop Sports Phys Ther. 1987;8: 567–573. 18797021 10.2519/jospt.1987.8.12.567

[34] Kraemer WJ, Patton JF, Gordon SE, Harman EA, Deschenes MR, Reynolds K, et al Compatibility of high-intensity strength and endurance training on hormonal and skeletal muscle adaptations. J Appl Physiol. 1995;78: 976–989. 10.1152/jappl.1995.78.3.976 7775344

[35] Putman CT, Xu X, Gillies E, MacLean IM, Bell GJ. Effects of strength, endurance and combined training on myosin heavy chain content and fibre-type distribution in humans. Eur J Appl Physiol. 2004;92: 376–384. 10.1007/s00421-004-1104-7 15241691

[36] Kikuchi N, Yoshida S, Okuyama M, Nakazato K. The effect of high-intensity interval cycling sprints subsequent to arm-curl exercise on upper-body muscle strength and hypertrophy. J Strength Cond Res. 2016;30: 2318–2323. 10.1519/JSC.0000000000001315 26694501

